# A high-precision oasis dataset for China from remote sensing images

**DOI:** 10.1038/s41597-024-03553-0

**Published:** 2024-07-02

**Authors:** Jingwu Lin, Dongwei Gui, Yunfei Liu, Qi Liu, Siyuan Zhang, Chuang Liu

**Affiliations:** 1 State Key Laboratory of Desert and Oasis Ecology, Key Laboratory of Ecological Safety and Sustainable Development in Arid Lands, Xinjiang Institute of Ecology and Geography, Chinese Academy of Sciences, Urumqi, 830011 China; 2 University of Chinese Academy of Sciences, Beijing, 100049 China; 3 Cele National Station of Observation & Research for Desert Grassland Ecosystem in Xinjiang, Cele, 848300 China; 4 Institute of Geographic Sciences and Natural Resources Research, Chinese Academy of Sciences, Beijing, 100101 China

**Keywords:** Environmental sciences, Space physics

## Abstract

High-resolution oasis maps are imperative for understanding ecological and socio-economic development of arid regions. However, due to the late establishment and relatively niche nature of the oasis discipline, there are no high-precision datasets related to oases in the world to date. To fill this gap, detailed visual interpretation of remote sensing images on Google Earth Professional or Sentinel-2 was conducted in summer 2020, and for the first time, a high-precision dataset of China’s oases (abbreviation HDCO) with a resolution of 1 meter was constructed. HDCO comprises 1,466 oases with a total area of 277,375.56 km^2^. The kappa coefficient for this dataset validated by the field survey was 0.8686 and the AUC value for the ROC curve was 0.935. In addition, information on the geographic coordinates, climatic conditions, major landforms, and hydrological features of each oasis was added to the attribute table of the dataset. This dataset enables researchers to quantitatively monitor location and area of oases, fosters exploration of the relationship between oases and human under climate change and urbanization.

## Background & Summary

Drylands are an important aspect of the Earth’s geographic composition, occupying more than 40 percent of the global land area^1,2^. These regions, known for their scarce precipitation and high evapotranspiration potential, are highly sensitive to global change drivers^3^. They are not only vulnerable to climate fluctuations^4^ but also pose a serious threat to human activity systems due to crises triggered by increased poverty, food insecurity, and regional political instability^5^. An oasis is a special landscape combining nature and humanity in drylands^6^. In these arid regions, occasional oases appear in the vast desert terrain that hosts most of the developing countries and marginalized populations^7^. An oasis is a non-zonal geographic unit on a desert substrate in an arid zone that is driven by a stable water source. It contrasts with the arid and barren environment by having abundant water, fertile soil, and lush vegetation. Oases are important centers of agriculture, pastoralism, and human activity in arid zones^8^. Therefore, as the core of the human-land system in the arid region, oases have become the key area of concern for scholars studying arid regions.

From the perspective of physical geography, revealing the spatial and temporal distribution characteristics of oases is the core content of oasis science. Clarifying the distribution location and area change of oasis is of great significance for scientific research in arid zones. Although there have been many research results on oases in individual regions^9,10^ or certain areas^11–13^, they are generally characterized by fragmentation and a lack of comprehensive and macroscopic understanding of oases on a large scale, such as the national and global scales. Unlike geographic units with detailed distribution datasets, such as glaciers^14,15^ and lakes^16,17^, no high-precision oasis-related datasets are available in the world to date. This has made the accurate distribution of oases plagued by unclear basic records and large data discrepancies, which have led to many contradictions and errors in numerous studies and hindered the development of the oasis discipline. Especially in the context of climate change, the problem of desertification has become increasingly serious. Therefore, clarifying the distribution of oases is crucial to realizing the land degradation neutrality of SDG15.3 target and the sustainable development of arid zones^18^. Therefore, conducting a comprehensive survey on the spatial distribution characteristics of oases and creating a dataset is both fundamental and pioneering work. It is of great value in consolidating the foundation of oasis science, describing the distribution of oases globally and finely, and filling the gaps in research related to oasis science.

China has the largest area of oases in the world, covering a wide range of zones with a variety of complex types. Oases play an important role in the arid regions of Northwest China, hosting 90% of the population and providing 95% of the economic output^19,20^. Clarifying the distribution of oases in China not only clarifies the current status of oases in China, but also helps to promote the development of global oasis research. Therefore, this paper provides a high-precision dataset covering all the oases in China, with 2020 as the base year. This dataset captures the spatial attributes and natural characteristics of the oases, providing benchmark data for assessing the impact of climate change and land use changes on the oases. Additionally, it is conducive to the accurate, rapid, and innovative development of oasis research.

## Methods

### Construction of the oasis distribution dataset in China

**Fig. 1:**
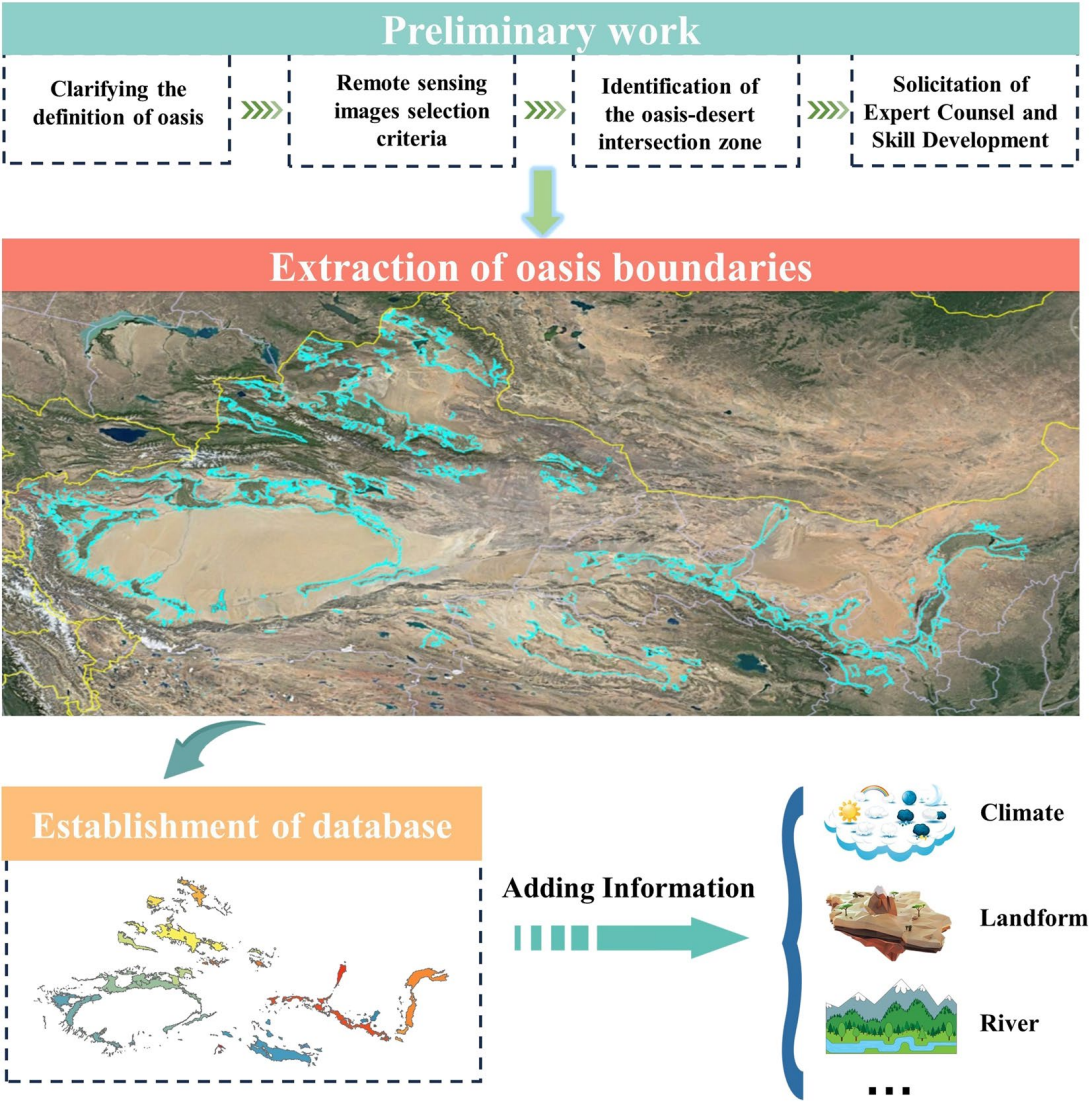
The technological progression of HDCO. HDCD: high-precision dataset of China’s oases.

In recent years, the rapid advancement of remote sensing technology has led to an increasing extent of coverage, a significant enhancement in the quality of remote sensing imagery, and a marked improvement in image clarity. Remote sensing images acquired through a variety of satellites and sensors furnish insights into the distinct attributes of ground objects, spanning across spectral, temporal, and spatial dimensions^21^. These data form an indispensable wellspring of information for scrutinizing the spatial expanse of oases^22^. Although a number of automated^23–25^ or semi-automated^26,27^ techniques have emerged for oasis delineation, most of these methods are confined to smaller areas and often produce unsatisfactory results. This limitation arises from the spatial heterogeneity of vegetation types within oases and the diversity of oasis boundary patterns. Consequently, manual intervention is often necessary to correct the resulting uncertainties^28^. In addition, the precise delineation of the oasis-desert boundary in the oasis-desert transition zone poses a great challenge to the automatic extraction method^29^, and at present, the only way to obtain an accurate and high-precision oasis dataset is through manual visual interpretation. Therefore, in this study, a visual interpretation approach characterized by enhanced consistency and rigorous quality control was opted for. Comprehensive extraction, validation, modification, and integration of oasis data from all over China was carried out by twelve skilled technicians divided into four different teams over a period of three years. The technological progression is shown in Fig. 1.

### Clarifying the definition of oasis

The collation of existing knowledge related to oases is undertaken, with a primary focus on consolidating the varying definitions attributed to the term “oasis.” The precise definition of an oasis serves as the bedrock for determining its spatial extent and delineating it from desert landscapes, but different researchers often have different definitions of oases based on different research purposes^30,31^. Through the comprehensive comparison of all aspects, combined with the characteristics of the oasis on remote sensing images and the field survey data, it is finally clear that the oasis must have the following three characteristics: (1) exist within arid and semi-arid areas; (2) be surrounded or semi-surrounded by a desert environment; (3) heterogeneous landscape units with a certain level of vegetation cover or economic ecological power, driven by a stable water source. This clear elucidation of the oasis concept not only establishes a theoretical framework for subsequent investigations but also lays the groundwork for addressing the challenges of oasis identification during information extraction processes.

### Remote sensing images selection criteria

Most of the image data used for oasis extraction came from Google Earth Pro, a dataset that is a product of multiple sets of satellite maps provided by different commercial image providers or government agencies at different zoom levels. Remote sensing image data on Google Earth Pro have a wide coverage, and optical sensors (e.g., QuickBird, Worldview) have high resolution down to the sub-meter level. The selection of images greatly impacts the accuracy of the extraction and the precision of the dataset, so strict adherence to the following criteria was maintained: (1) The year 2020 was more humid compared to previous years^32^, benefiting the growth of oasis vegetation. Summer, with its absence of snow cover, is the peak season for vegetation growth, offering the most pronounced contrast between the oases and the desert. This contrast makes it easier to distinguish between different types of land cover through image features.; (2) the cloud coverage in the images must be less than 5%, and the oasis boundary areas cannot be obscured by clouds; (3) for areas that have no or only low-quality images on Google Earth Pro in 2020 (e.g., high mountain edges and isolated rural areas), Sentinel-2 images with 10 m spatial resolution or images from similar years were used instead.

### Identification of the oasis-desert intersection zone

A pivotal endeavor within oasis boundary extraction involves the precise delineation of the transitional area between oases and deserts^33^. This narrow strip, functioning as a bridge between these two contrasting landscapes, bears the impact of both oasis development and desertification, simultaneously capturing the ebb and flow of oasis expansion over time^34^. Given its intrinsic resemblance to the oasis terrain, outlining its boundaries frequently becomes a subject of debate, thereby introducing complexities into the extraction process. The demarcation between oasis and desert becomes discernible with relative ease when the transition zone encompasses distinctive features like artificial structures, protective tree forests, and cultivated lands. Nonetheless, it is difficult to distinguish between transition zones if they are areas with some but not much vegetation cover, such as naturally vegetated areas on river floodplains, agricultural land that has not yet been fully retired, or wetlands in deserts. Addressing this intricacy is a matter that merits thorough exploration. After iterative experimentation, a definitive approach was arrived at. Land usage is classified based on remote sensing imagery in our strategy, and subsequently, Fractional Vegetation Cover (FVC) is calculated. This approach has culminated in a technical trajectory that successfully addresses this intricate task.

First, a set of training samples from the designated region was curated for evaluation. The supervised classification of the remotely sensed images was performed using ENVI software, categorizing the land use types into six main groups: agricultural land, man-made structures, forest, water bodies, grass/shrubs, and desert. Next, the normalized vegetation index value (NDVI) was calculated for each image element of the type using the formula^35^:

$${\rm{NDV}}I=\frac{{\rm{NIR}}-{Red}}{{NIR}+{Red}}$$

where NIR is the near-infrared band and Red is the infrared band.

Then, after acquiring the NDVI value, the Fractional Vegetation Cover is calculated using the subsequent equation^36^:

$${FVC}=\frac{N{\rm{DV}}I-{NDV}{I}_{{soi}}}{{NDV}{I}_{{veg}}-{NDV}{I}_{{soi}}}\times 100$$

where NDVI_soi_ and NDVI_veg_ are the observed values at bare soil (vegetation cover is 0) and full canopy cover (vegetation cover is 100%), respectively.

Ultimately, the yielded FVC value lies within the range of 0 to 1. The image elements with FVC values below 0.2 in arid zones and below 0.3 in semi-arid zones are categorized as desert regions, while those surpassing this threshold are designated as oasis zones. This categorical differentiation ensures a stringent demarcation between the two distinct zones.

### Solicitation of expert counsel and skill development

To mitigate discrepancies resulting from inconsistent standards in delineating oasis boundaries and variable levels of operator expertise, a panel was convened before the project began. The group, composed of experts in oasis science, remote sensing image recognition and data classification, developed specifications and operational guidelines for extracting oasis data. The specifications establish explicit criteria for identifying land cover types within remote sensing imagery, provide comprehensive documentation protocols for the oasis extraction process and its outcomes, and offer precise guidelines for managing oasis boundary data through Geographic Information Systems. The operational guide includes detailed steps on how to download and open Google Earth Pro, along with a comprehensive introduction to the software’s basic functionalities. It also includes instructions on how to draw boundary lines during the oasis extraction process, how to annotate when the feature type of the image is not clear, and so on. Moreover, each oasis extractor underwent expert guidance and proficiency training before undertaking the task of visual interpretation. Experts led the trainees through field surveys to explain the differences between actual land features and their representations in remote sensing imagery, highlighting characteristics such as shape, size, shading, texture, and color. Subsequently, the trainees underwent proficiency tests in five designated experimental areas. They were required to accurately identify oasis areas based on the decoding signs in the remote sensing images. Only after mastering these skills were, they allowed to formally commence oasis extraction operations.

### Oasis boundary vectorization

Oasis vectorization extraction was conducted by a dozen geography-related workers during 2020–2023. For the oasis extraction, the images on Google Earth Pro were zoomed to the maximum (view height less than 1.5 km and spatial resolution less than 1 m) to determine the feature type and set control points based on ground truth using the mapping function. When the edge of the oasis is an irregular feature types (such as forest, wetland, water body, etc.), the interval between two control points should not exceed 1 m. Conversely, for more regular feature type (such as farmland, factory, road, etc.), the interval between two control points can be appropriately lengthened, but it should not exceed 5 m at most. When the terrain is more complex, terrain data is used as a reference, and the 3D visualization function of Google Earth Pro is employed to observe the surface from different angles to assist in image interpretation. In instances where judgments posed exceptional difficulty grounded solely in satellite imagery, a multifaceted approach was employed. Latitude and longitude coordinates were documented for such enigmatic locations, and subsequent deliberation within the team, engagement with local experts, and on-site field investigations collectively determined the classification of these locations within or outside the oasis area.

### Validation of oasis vectorization results

**Fig. 2:**
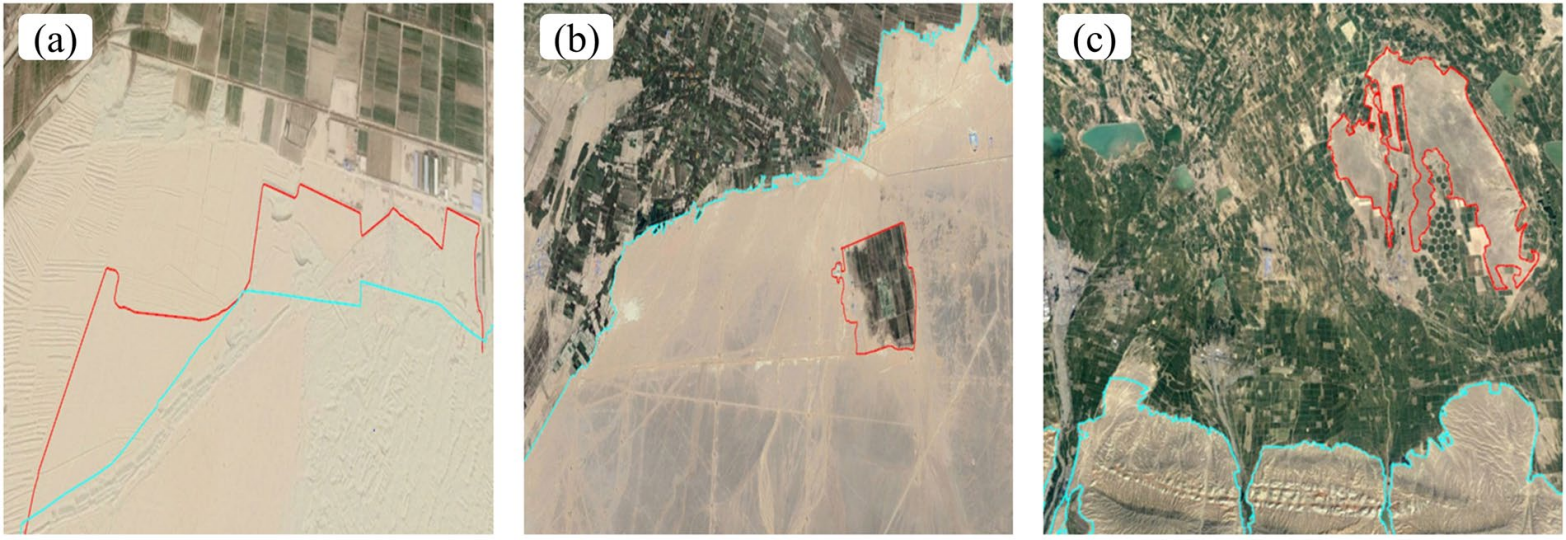
Schematic diagram of the Oasis vectorization work modification. Blue is the uncorrected border and red is the corrected or newly added border. **(a)** represents omission of smaller oases with smaller areas next to large oases that are easily overlooked. **(b)** represents omission of smaller oases with smaller areas next to large oases that are easily overlooked. **(c)** represents failure to remove desert parts interspersed in the oases.

The assessment of extraction outcomes encompasses three distinct methodologies: expert sampling, interactive inspection, and field survey. Expert sampling is carried out after the completion of the mapping of a large area of an oasis, with a number of small areas randomly selected by relevant experts in data publishing to ensure that the accuracy of the mapping meets the requirements and that the boundaries of the oasis are accurately delineated. Interactive inspection entails the transmission of vectorized extraction results from one group to another for scrutiny. Subsequently, a third group arbitrates over any discrepancies concerning oasis boundaries that were extracted by the initial two groups. Field investigation then involves fieldwork at the locations that are disputed during extraction, using cameras and drones to capture images from varying angles and heightened resolutions and to record the types of features. There are three main types of errors in the vectorized extraction process: (1) misjudgment of oasis boundaries and deviations between the outline of oasis boundaries and the actual situation (Fig. 2a); (2) omission of smaller oases with smaller areas next to large oases that are easily overlooked (Fig. 2b); and (3) failure to remove desert parts interspersed in the oases (Fig. 2c). If the area of open space in an oasis exceeds 36 pixels, it is considered a desert and should be removed; otherwise, it will result in an overestimation of the area of that oasis when calculating its size.

### Oasis database establishment

The database construction unfolds through a sequential process. Firstly, KMZ files generated on Google Earth Pro are imported into ArcGIS, where they are subsequently converted into vector files in shp format. Subsequently, meticulous examination of spatial topological relationships commences, primarily aimed at bridging suspension points, eliminating superfluous line segments, and rectifying potential polygon geometry inconsistencies. Moreover, polyline files are transmuted into polygon files, with an established minimum oasis size of 0.01 km² employed to expunge inadequately sized patches. This results in the creation of spatially distinct, self-contained block oases, culminating in a preliminary dataset of Chinese oases. Following this, the dataset’s spatial projection is transformed into the Albers Orthographic Equivalent Standard Latitudinal Cut Cone Projection (Central Longitude 91°E, Standard Latitudes 35°N and 49°N, WGS84 Coordinate System) for the systematic categorization and area computation of Chinese oases. Finally, the shp files are reverted to Google Earth Pro, and each oasis undergoes meticulous scrutiny to ensure that no alterations in shape or information omission occurred during the data format conversion and topology processing.

### Enrichment of the HDCO

In order to better obtain comprehensive information about China’s oases and increase the richness of HDCO, this paper enhances the attribute table of the dataset by seamlessly integrating publicly available data sources. This integration is achieved through spatial analysis and mathematical-statistical methods to more accurately characterize the geographic attributes of each oasis. This integration involved the amalgamation of publicly accessible datasets pertaining to administrative regions, river networks, climatic conditions, and elevation information. Despite originating from disparate sources, these datasets shared a common spatial context, which facilitated their synchronization. Specifically, information pertaining to provincial administrative boundaries and the distribution of prominent rivers in China was procured from the website of the National Basic Geographic Information Center (http://www.ngcc.cn/). Elevation data was extracted form DEM data, which were obtained from the ALOS satellite 12.5 m product of the Japan Aerospace Research Institute (https://search.asf.alaska.edu/). Climate data were acquired from the Resource and Environment Science and Data Center of the Chinese Academy of Sciences (https://www.resdc.cn/). During the course of this study, these datasets, available in both vector and raster formats, were linked to the oasis dataset on a one-to-one basis. This linkage was accomplished utilizing Geographic Information System (GIS) tools through two primary methodologies: spatial connectivity and regional statistics. The spatial connectivity tool can connect attributes from one feature to another through topological relationships of geospatial locations. This methodology is instrumental in delineating climatic zones, geomorphological units, river basins, and other pertinent characteristics pertaining to each oasis’s location. On the other hand, regional statistics enable analyses encompassing all image elements residing within each input region. This analytical approach generates statistical insights into the values encapsulated by raster data within the designated region. For instance, the average elevation was computed using this technique.

## Data Records

The HDCO is available at the Science Data Bank (https://www.scidb.cn/s/2iEN3m)^37^. In addition, uncoded oasis data for more than 40 important regions in China have been released, and researchers who need the precise extent of oases in a particular region can download the data from Global Change Research Data Publishing & Repository (https://geodoi.ac.cn/)^38–79^.

**Fig. 3:**
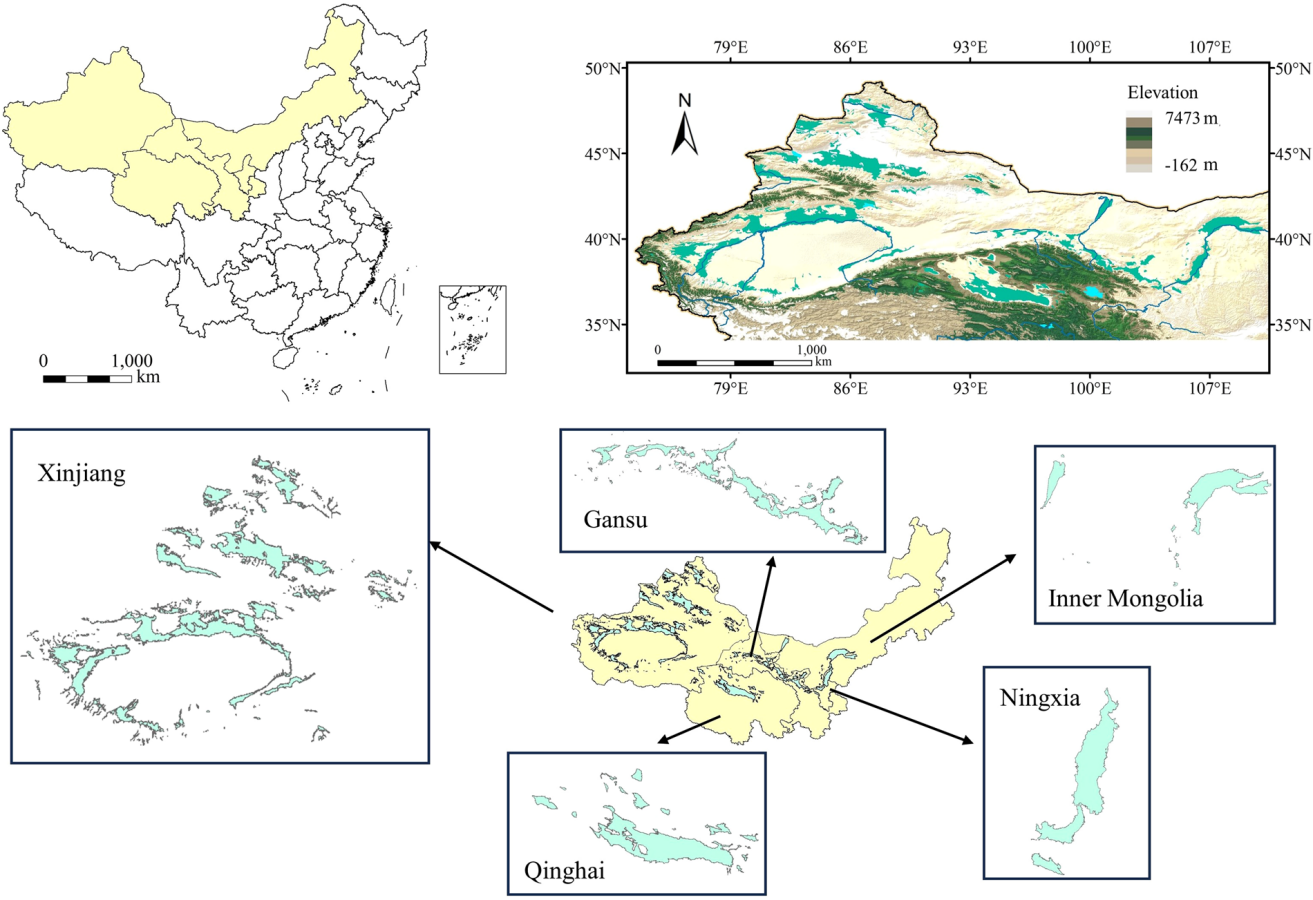
Spatial distribution of oases in China.

The distribution characteristics of oases in China in HDCO include a total of 1,466 oases with a total area of 277,375.56 km², accounting for about 3.02% of China’s area and 8.12% of China’s dryland area. As illustrated by Fig. 3, the geographical distribution of Chinese oases is delineated within the longitudinal span of 93.49°E to 104.69°E and the latitudinal interval of 36.36°N to 40.99°N. These oases are predominantly situated across the regions of Xinjiang, Gansu, the northwestern sectors of Qinghai, the northern precincts of Ningxia, as well as the central and western expanses of Inner Mongolia. This distribution extends westward to encompass the Kashgar vicinity, stretches eastward to encapsulate Baotou, reaches northward to converge with the Altai Mountains, and delves southward to encompass the Kunlun Mountains and Qilian Mountains.

The attribute table of HDCO includes the following fields:

OasisID: The OasisID is constructed by amalgamating five paramount attributes that encapsulate the essence of the oasis: administrative region, climate zone, landform, river and area. The OasisID is a unique identifier for each oasis in the dataset, allowing each oasis to be accurately located and its geographic features displayed.

ProvinceID: Based on the latest data from the Chinese province-level division, the administrative regions of each oasis can be obtained, including Xinjiang (XJ), Gansu (GS), Qinghai (QS), Ningxia (NX), and Inner Mongolia (IM).

ClimateID: The climate zones in which the Chinese oases are located include Middle Temperate Zone (M), Warm Temperate Zone (W), and Plateau Climate Zone (P).

LandformID: Aligned with the Chinese landform zoning system^80^, the area containing each Chinese oasis is categorized into 23 tertiary landform zones. Table 1 provides a comprehensive depiction of the landform numbers in the oasis ID and the codes associated with the Chinese landform zoning system.

**Table 1:** Comparison of landformID and Chinese landform zoning scheme.

LandformID	Chinese landform zoning scheme
A	Alxa plateaus, hills, aeolian plains small-region
B	Mazong Mt middle mountains and hills small-region
C	Hexi Corridor alluvial-diluvial plains small-region
D	Turpan-Hami alluvial-diluvial plains small-region
E	Ulungur and Ertix rivers alluvial plains small-region
F	Gurbantunggut Desert small-region
G	Western Junggar middle mountains and hills small-region
H	Southern margin of Junggar Basin diluvial-alluvial plains small-region
I	Tianshan Mt north piedmont low mountains, hills and plains small-region
J	Eastern Tianshan Mt high mountains small-region
K	Central Tianshan Mt high mountains and basins small-region
L	Yanqi Basin small-region
M	Southern Tianshan Mt high mountains small-region
N	Northern Tarim River lacustrine-alluvial plains small-region
O	Southeastern margin of Tarim River alluvial-diluvial platforms and plains small-region
P	Southern margin of Tarim River alluvial-diluvial plains small-region
Q	Kashgar diluvial-alluvial plains small-region
R	Qaidam Basin small-region
S	Western segment of central Kunlun Mt high mountains and lake basins small-region
T	Western Kunlun Mt high and extremely high mountains small-region
U	Hetao alluvial plains small-region
V	Liupan Mt middle and low mountains, hills and valleys small-region
W	Gansu middle mountains, loessic ridges and mounds small-region

RiverID: An oasis is usually nourished by one or more rivers, and the attribute table shows the most important class 5 or higher rivers for each oasis. The OasisID river part is coded with reference to the newly released eight-digit code for Chinese river code^81^. Table 2 shows the detailed description of river names, Chinese river codes, and river numbers in the Oasis ID.

**Table 2:** Comparison table of river names, Chinese river codes and RiverID.

River name	Chinese River code	RiverID	River name	Chinese River code	RiverID
Yellow River	ADA00000	01	Kashgar River	AKL10106	43
Shule River	AKH10002	02	Gezi River	AKL10126	44
Party River	AKH10106	03	Chachemak River	AKL10156	45
Yulin River	AKH11002	04	Yarkant River	AKL10206	46
Oil River	AKH12002	05	Tashkurga River	AKL10216	47
Baiyang River (Hexi Corridor)	AKH12106	06	Tiznav River	AKL10236	48
Heihe River	AKH13002	07	Aksu River	AKL10306	49
Beida River	AKH13106	08	Toshkent River	AKL10316	50
Maying River	AKH13216	09	Hetian River	AKL10406	51
Shandan River	AKH13306	10	Taylvichuk River	AKL10536	52
Hong Shui River	AKH13336	11	Karasu River	AKL10546	53
Shiyang River	AKH16002	12	Kushan River	AKL11002	54
Fengle River	AKH20002	13	Uruk River	AKL14002	55
Yuka River	AKJ18002	14	Pishan River	AKL15002	56
Golmud River	AKJ19002	15	Sangzhu River	AKL16002	57
Bayingole River	AKJ58002	16	Celle River	AKL18002	58
Qaidam River	AKJ22002	17	Kriya River	AKL19002	59
Nur River	AKJ50206	18	Nya River	AKL20002	60
Ulungur River	AKK10002	19	Cherchen River	AKL21002	61
Habukesel River	AKK11002	20	Ruoqiang River	AKL22002	62
Baiyang River (Tower City)	AKK12002	21	Kuqa River	AKL24002	63
Dabult River	AKK13002	22	Kyzylkotan	AKL24306	64
Willow Gully River	AKK14002	23	Dina River	AKL25002	65
Quitten River	AKK17002	24	Kaidu River	AKL26002	66
Gurtu River	AKK17216	25	Peacock River	AKL27002	67
Four Trees River	AKK18002	26	Ulastai River	AKL28106	68
Bortala River	AKK19002	27	Buzang River	AKL55002	69
Daheyanzi River	AKK19206	28	Yatunguz River	AKL64002	70
Urtaxaray River	AKK19306	29	Bostantogerrak River	AKL65002	71
Manas river	AKK21002	30	Moleche River	AKL66002	72
Urumqi River	AKK26002	31	Karamilan River	AKL68002	73
Alla Ditch	AKK27002	32	Tashsayi River	AKL74002	74
Baiyang River (Fukang)	AKK28002	33	Gulgahd River	AKL86002	75
West Dalongkou River	AKK29002	34	Atat Atkan River	AKL88106	76
Coal kiln Ditch	AKK33002	35	Ixiekpati River	AKL90002	77
Daheyan River	AKK34002	36	Tailan River	AKL95002	78
Kokoyar River	AKK35002	37	Yangxia River	AKLA2002	79
Ertang Ditch	AKK36002	38	Irtysh River	AJG00001	80
Igo River	AKK39002	39	Emin River	AJF10001	81
Muhurtai River	AKK47002	40	Ili River	AJF11001	82
Shichengzi River	AKK71002	41	Muzart River	AJF11146	83
Tarim river	AKL10002	42			

AreaID: The size of an oasis. Within ArcMap, the WGS84 coordinate system is employed, quantified in square kilometers, and rounded to two decimal places.

Perimeter: The perimeter of the oasis, calculated in the same way as the oasis area.

Longitude: The X-coordinate denoting the center point of each oasis.

Latitude: The Y-coordinate designating the center point of each oasis.

Mean_Elev: The average elevation value of each oasis, serving as a representative measure of its elevation.

Min_Elev: The minimum elevation of the oasis.

Max_Elev: The highest elevation attained within the oasis.

Dif_Elev: This value signifies the discrepancy between the highest and lowest elevations within the oasis.

Basin: The Chinese oases are located in five basins, including the Yellow River mainstream basin, the Hexi Corridor-the Alxa inflow zone, the Qaidam inflow zone, the Junggar inflow zone, the Tarim inflow zone, the Irtysh River basin, and the Ili-Emin River basin.

## Technical Validation

Despite rigorous adherence to the principles and norms of image interpretation work during the production of HDCO, some imperfections due to both subjective and objective factors were inevitable. Subjective sources of imperfections included variations in personnel proficiency levels, the extent of familiarity with the study area, and experience in executing vectorization operations. Objective sources of imperfections could be attributed to the varying quality of remote sensing images due to the expansive geographical scope of the study area. Moreover, disparities stemming from image data collected by different sensors and at different chronological moments contributed to these imperfections. Therefore, the accuracy of the dataset was validated using field surveys and random hexagonal grids.

### Field survey assess accuracy

**Fig. 4:**
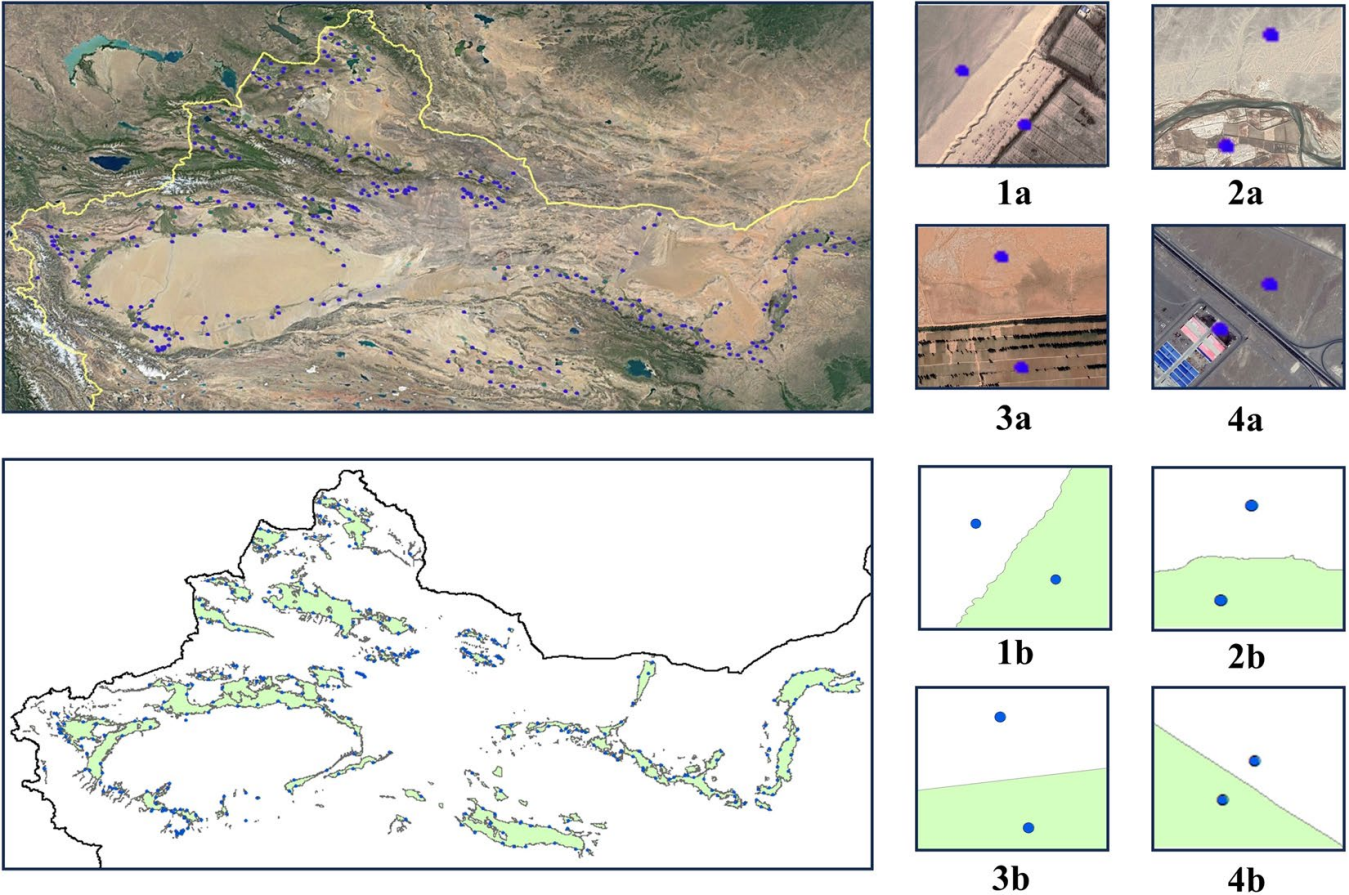
An overview of the validation point location map and examples of detailed information of validation point. **1a-4a** are points obtained from the field survey. **1b-4b** are the corresponding points in the dataset.

Between June 2020 and August 2023, a comprehensive field survey was conducted in the oasis distribution areas of China. Before departure, a detailed field survey plan was formulated, covering the survey routes, sample point distribution, and the necessary equipment. Particular emphasis was placed on the selection of sample points, considering their representativeness and statistical distribution standards. To ensure the scientific rigor and comprehensiveness of the sample points, the method focused on selecting points that covered the entire geographical range, were evenly distributed, and exhibited distinct and identifiable features during the selection process. During the field survey, precise symmetrical measurements were conducted within a 10-meter buffer zone at each sample point using GPS equipment. This method ensured that each measurement point corresponded to an oasis area, while its paired point represented a non-oasis area. Simultaneously, drone aerial photography technology was employed to obtain high-resolution images, supplementing the ground measurements. Moreover, environmental information such as vegetation types, coverage, and soil types were recorded for each sample point as comprehensively as possible. These data are crucial for understanding the mechanisms of oasis boundary formation and changes. Ultimately, using high-precision global positioning system technology, 700 precise points were obtained at 350 locations, as shown in Fig. 4.

Due to various factors, there are partial inconsistencies between the remote sensing data collection times used by HDCO and the GPS measurement times during field surveys. To meet this challenge, Google Earth Pro or Sentinel-2 images were selected that best matched the timing of GPS measurements in the field. The oasis boundaries of the remotely sensed images were re-extracted for validation using the same methods and steps as before. Thus, the time of the remote sensing image and the time of the field survey verification point collection were consistent to ensure the reliability and accuracy of the verification point.

The dataset’s accuracy was evaluated by conducting a comparative analysis between the validation points derived from field measurements and those within the HDCO. Confusion matrices and Kappa coefficients were employed in the assessment. As shown in Table 3, the overall accuracy (OA) of the mapped areas and validation points was 93.43%. The producer accuracy (PA) of the oasis class was 95.04% and the user accuracy (UA) was 91.83%. The producer accuracy (PA) for the non-oasis class was 91.88% and user accuracy (UA) was 95.07%. The Kappa coefficient is often used as a consistency test and can be a good measure of overall classification performance^82^. The final calculated result was 0.8686, indicating excellent performance of the dataset validation accuracy.

**Table 3:** Confusion matrix and Kappa coefficients.

Field\Dataset	Oasis	No-oasis	Total	UA
Oasis	326	29	355	91.83%
No-oasis	17	328	345	95.07%
Total	343	357	700	
PA	95.04%	91.88%		
OA	93.43%			
Kappa	0.8686			

PA: producer accuracy. OA: overall accuracy. UA: user accuracy.

**Fig. 5:**
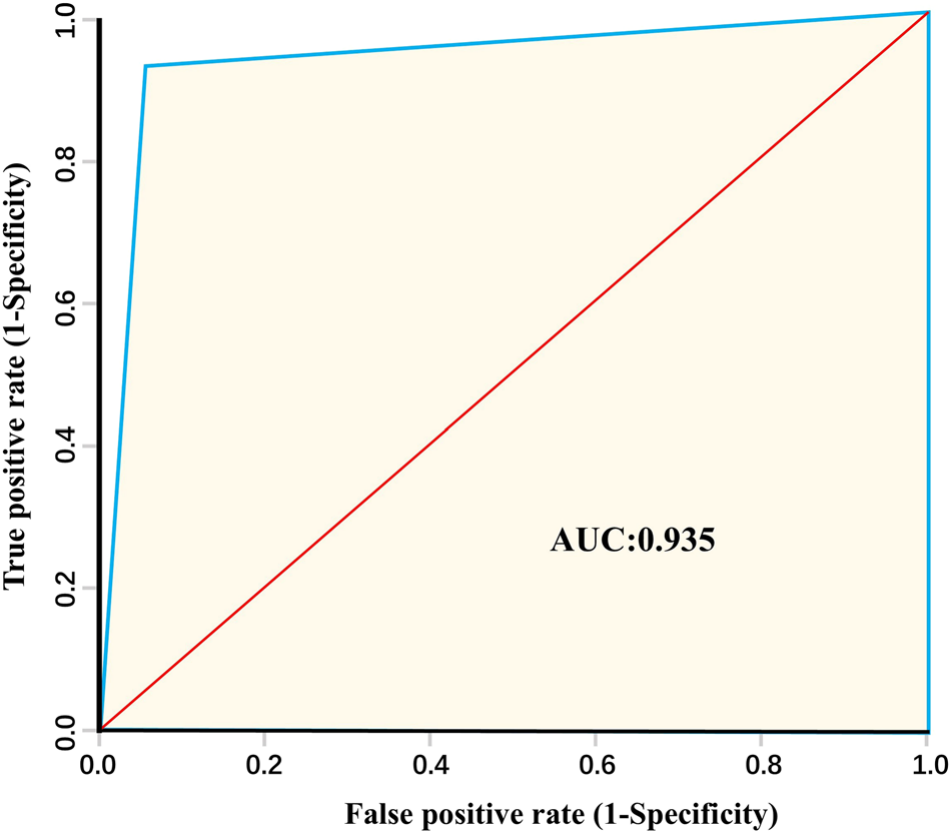
ROC graph. ROC: Receiver Operating Characteristic. AUC: Area Under the Curve.

The receiver operating characteristic (ROC) curve is a good method for visualizing accuracy. It is a curve based on a series of different dichotomous classifications with the true positive rate (sensitivity) as the vertical coordinate and the false positive rate (1-specificity) as the horizontal coordinate. The area enclosed by the ROC curve and the specificity is called the AUC value and takes a value between 0 and 1. A value closer to 1 indicates a better test result. The AUC value of 0.935 in Fig. 5 indicates that the dataset is highly accurate, with only 6.65% of the oasis areas not plotted or non-oasis areas misclassified as oasis.

### Accuracy assessment from random hexagonal grid

**Fig. 6:**
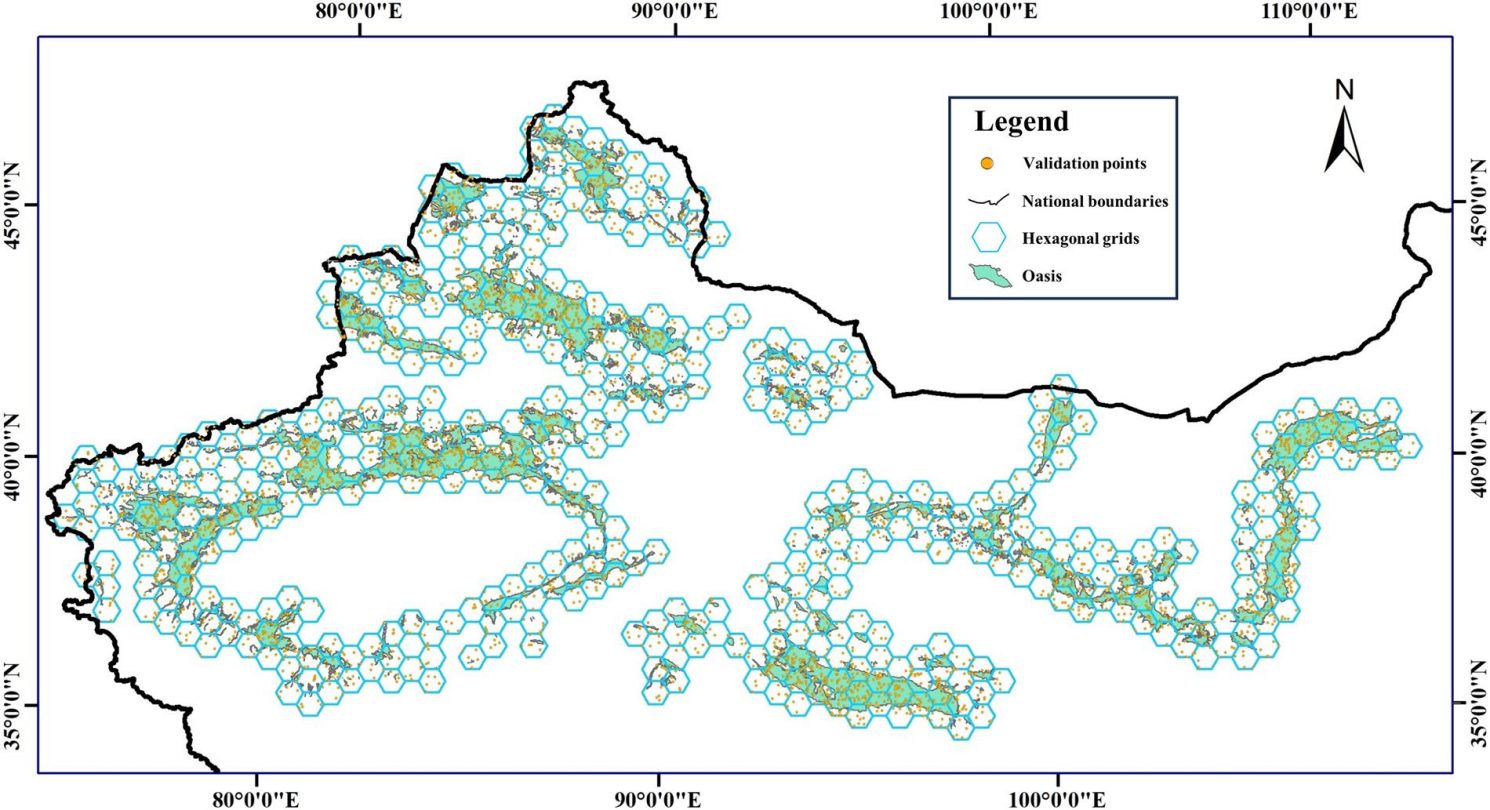
Distribution of hexagonal grids and validation points.

While accuracy verification through field surveys offers a high degree of confidence, it is time-consuming and challenging to verify every oasis, particularly the smaller ones, due to the constraints of limited sampling points. To complement this approach, an alternative method commonly employed for verifying the accuracy of remote sensing classification was utilized. A hexagonal grid measuring 50,000 × 50,000 meters was established to cover all oasis areas across China. Subsequently, random points were generated based on the proportion of oasis area within each hexagonal grid cell (as illustrated in Fig. 6): 0%–20% represented by 5 points, 20%–40% by 10 points, 41%–60% by 15 points, 60%–80% by 20 points, and 80%–100% by 25 points. After the latitude and longitude coordinates of each point were extracted and it was determined whether they fell within the dataset’s designated oasis areas, these points were imported into Google Earth to visually confirm whether they were truly oases. Eventually, 519 hexagonal grids and 5,549 points were generated, of which 5,342 points were correct, resulting in an accuracy of 96.27%.

## Usage Notes

Geographic information system (GIS) software such as ArcGIS and QGIS is recommended for accessing and processing data to visualize the distribution of oases in China and to process the data. HDCO’s attribute table provides a wealth of information, including oasis identification, geographic location, area, climate, geomorphology, and rivers. The dataset is useful for analyzing the various factors affecting the formation and development of oases. For example, the dataset represents the current distribution of oases in China under the current climate conditions and can be used to simulate the distribution pattern of oases under future climate change scenarios through machine learning models. This dataset will be an important tool for oasis scholars, climatologists, ecologists, and others, and will provide a reference for subsequent research on oasis datasets.

## Data Availability

The final results of this paper, which utilizes a random hexagonal grid to validate the accuracy of the HDCO, have been published on GitHub and can be accessed at https://github.com/Linjingwu999/HFOCD.git. This includes the code used, the generated hexagonal vector grid, the percentage of area occupied by each grid oasis, and the location of the validation points. Python 3.9 should be used to access and edit the code.
